# Does Preendoscopy Rockall Score Safely Identify Low Risk Patients following Upper Gastrointestinal Haemorrhage?

**DOI:** 10.1155/2015/410702

**Published:** 2015-05-18

**Authors:** Matthew R. Johnston, Iain A. Murray, Michael Schultz, Peter McLeod, Nathan O'Donnell, Heather Norton, Chelsea Baines, Emily Fawcett, Terry Fesaitu, Hin Leung, Jeong-Yoon Park, Adibah Salleh, Wei Zhang, José A. García

**Affiliations:** ^1^Department of Preventive and Social Medicine, Dunedin School of Medicine, University of Otago, Dunedin 9054, New Zealand; ^2^Gastroenterology Unit, Southern District Health Board, Dunedin Hospital, 201 Great King Street, Dunedin 9016, New Zealand; ^3^Department of Gastroenterology, Royal Cornwall Hospital, Truro, Cornwall TR1 3LJ, UK; ^4^Department of Medicine, Dunedin School of Medicine, University of Otago, unedin 9054, New Zealand

## Abstract

*Objective.* To determine if preendoscopy Rockall score (PERS) enables safe outpatient management of New Zealanders with upper gastrointestinal haemorrhage (UGIH).* Methods.* Retrospective analysis of adults with UGIH over 59 consecutive months. PERS, diagnosis, demographics, need for endoscopic therapy, transfusion or surgery and 30-day mortality and 14-day rebleeding rate, and sensitivity and specificity of PERS for enabling safe discharge preendoscopy were calculated.* Results.* 424 admissions with UGIH. Median age was 74.3 years (range 19–93 years), with 55.1% being males. 30-day mortality was 4.6% and 14-day rebleeding rate was 6.0%. Intervention was required in 181 (46.6%): blood transfusion (147 : 37.9%), endoscopic intervention (75 : 19.3%), and surgery (8 : 2.1%). 42 (10.8%) had PERS = 0 with intervention required in 15 (35.7%). Females more frequently required intervention, OR 1.73 (CI: 1.12–2.69). PERS did not predict intervention but did predict 30-day mortality: each point increase equated to an increase in mortality of OR 1.46 (CI: 1.11–1.92). Taking NSAIDs/aspirin reduced 30-day mortality, OR 0.22 (CI: 0.08–0.60).* Conclusion.* PERS identifies 10.8% of those with UGIH as low risk but 35.7% required intervention or died. It has a limited role in assessing these patients and should not be used to identify those suitable for outpatient endoscopy.

## 1. Introduction

Acute upper gastrointestinal haemorrhage (UGIH) is a common presentation to emergency departments throughout the world, with an estimated incidence in Europe and North America of 20–60/100,000 adults per year [1–3]. The severity of bleeding varies markedly and 80% will settle spontaneously without intervention [4]. Mortality averages 10–14% [3–7], with little improvement over the past 30 years.

Stratifying patients to determine low risk patients can allow discharge from the emergency department with planned outpatient endoscopy [8–16]. Glasgow Blatchford score (GBS) is probably the commonest used system and generally has high sensitivity and specificity for identifying low risk patients, although the percentage of low risk patients does vary in European and Asian studies from 7.9–34.2% [9–13, 15] and the optimum score varies from 0 to <2, with and without age modification. In our patients a GBS of <1 identified only 3.6% of patients as low risk with 100% sensitivity but 6.9% specificity [17]. Such low rates have led some observers to state that the risk of outpatient management is not warranted by the number of admission days saved [14].

Others have suggested that the full Rockall score (which requires endoscopy) and the preendoscopy Rockall score (PERS) may be superior to GBS in predicting more low risk patients who can safely be managed in the community, and PERS is easier to calculate [18]. Sometimes only full Rockall and not GBS predicts outcome, specifically rebleeding and mortality [19]. An Asian-Pacific working group has advocated admission and early assessment using preendoscopy prognostic scales and early discharge if there is low risk for recurrent bleeding and if there are no comorbidities [20].

Dunedin Hospital is a 388-bed teaching hospital in New Zealand's South Island and serves both urban and rural populations, covering a radius of 300 kilometres, serving 180,000. All upper gastrointestinal endoscopies are performed by one of 4 consultant gastroenterologists or a registrar under direct supervision and high risk lesions (active arterial bleeding, nonbleeding visible vessels, or adherent clot) are treated endoscopically. The hospital has guidelines to admit all patients presenting with UGIH and strict criteria for blood transfusion.

We have previously reported on patients presenting with UGIH and used variations of GBS to determine safety and practicality of immediate discharge for outpatient endoscopy, comparing them with an international cohort [17]. In these same patients, we have retrospectively analysed clinical, biochemical, and haematological parameters and determined whether PERS could successfully identify more patients than GBS.

## 2. Methodology

A retrospective review of medical records was approved by the Lower South Regional Ethics Committee. Patients who had a gastroscopy with indication of haematemesis or melaena between 01 January 2007 and 23 November 2011 at Dunedin Hospital were identified using our in-house electronic endoscopy database (Endosmart).

Based on the frequency of endoscopic therapy, transfusion, and surgery from previous studies we estimated a sample size of 400–600 was required to reliably determine if GBS or PERS could safely identify low risk patients.

If patients were referred as outpatients from general practice and had their gastroscopy without inpatient assessment, they were excluded. They were also excluded if they had an UGIH during an admission for another diagnosis or if their admission had been precipitated for symptoms not suggestive of acute UGIH. Patients under 16 years of age were also excluded. Multiple presentations by a single patient were included unless the admission was within 14 days of the index presentation that is defined as a “rebleed”.

The primary outcome was requirement for any intervention (endoscopic, surgery, or blood transfusion). Secondary outcomes included 30-day mortality and 14-day readmission, for any reason including rebleeding. Patients were considered high risk for outpatient management if they fulfilled any primary or secondary outcomes and low risk if not. Endoscopic therapy was reserved for those with stigmata of recent haemorrhage and blood transfusion for those with haemoglobin concentration below 90 g/L and symptomatic.

Demographics, comorbidity, admission medication, endoscopy diagnosis, clinical, biochemical, and haematological data, and 14-day rebleeding and readmission and 30day mortality were determined from electronic and paper patient records.

PERS was calculated for each patient admission and sensitivity, specificity, and positive and negative predictive values of PERS = 0 were calculated. Separate analyses were performed for the primary outcomes measures and for 30-day mortality and 14-day readmission.

A logistical regression model was used to determine the effect of PERS on the need for intervention while adjusting for possible confounders including sex, age (whether over 70), proton pump inhibitor (PPI) preadmission, nonsteroidal anti-inflammatory drug (NSAID) or aspirin preadmission, and symptom duration (under 24 hours, 1–7 days, or greater than 7 days). The model was simplified by the removal of nonsignificant parameters. Similar models were performed for 30-day mortality and 14-day readmission. A *p* value <0.05 was considered significant. The software used for logistic regression was R, version 2.14, a statistical computing and graphics package [21].

## 3. Results

**Figure 1:**
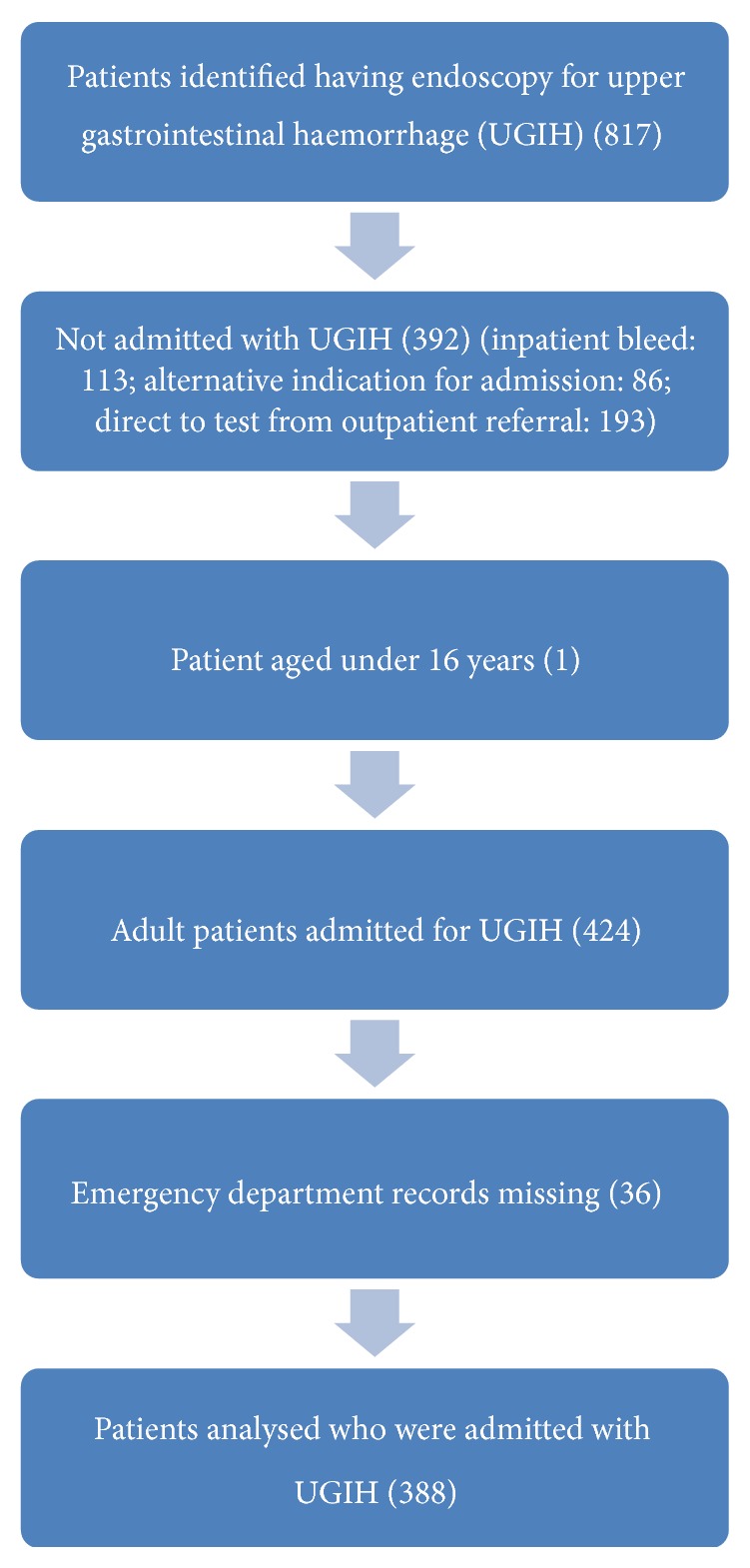
Flow chart demonstrating identification of patients relevant to this study, that is, adult patients admitted with a primary diagnosis of upper gastrointestinal haemorrhage.

Of 817 patients who had a gastroscopy for UGIH, 424 were analysed but data was incomplete in 36: in almost all cases the emergency department records were missing which included initial pulse and BP recordings and hence neither GBS nor PERS could be calculated. The reasons for excluding others are shown in Figure 1. 214 (55.1%) were male, median age 74.3 years (range 19.1–93.2 years). 376 were admitted directly to Dunedin Hospital with 12 transferred from smaller district hospitals.

Clinical details are given in Table 1, including presenting symptom (melaena, haematemesis and its nature, or both), symptom duration from first symptom to presentation in the emergency department. The majority of cases were New Zealand European or other Europeans (93.6%), with others of Maori (2.1%), Pacific Island (1.6%), Asia (1.0%), Africa (0.3%), and unidentified origin (1.6%).

As previously reported [17] the most common findings were gastritis, duodenitis, or oesophagitis (43.0%), peptic ulcers (35.3%), gastric ulcer (17.5%), normal (11.9%), oesophageal or gastric varices (4.8%), and malignancy (3.1%).

Of the 181 cases (46.6%) that had an intervention, 75 (19.3%) had endoscopic therapy, 147 (37.9%) had a blood transfusion, and 8 (2.1%) underwent surgery.

30-day mortality was 4.6% (18 patients) and 14-day rebleed incidence was 6.0%.

A complete list of outcomes is shown in Table 2.

**Figure 2:**
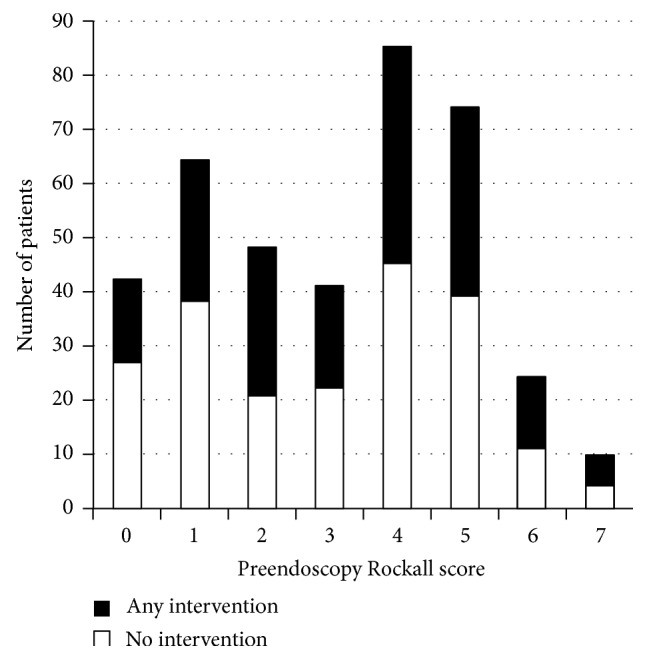
Preendoscopy Rockall score (PERS) for 388 consecutive patients admitted with upper gastrointestinal haemorrhage over 5 years (2006–2011). The need for any intervention (endoscopic, surgical, or blood transfusion) is shown. The PERS range of the study population was 0–7 with an average of 3.11 : 46.7% of patients required intervention.

**Figure 3:**
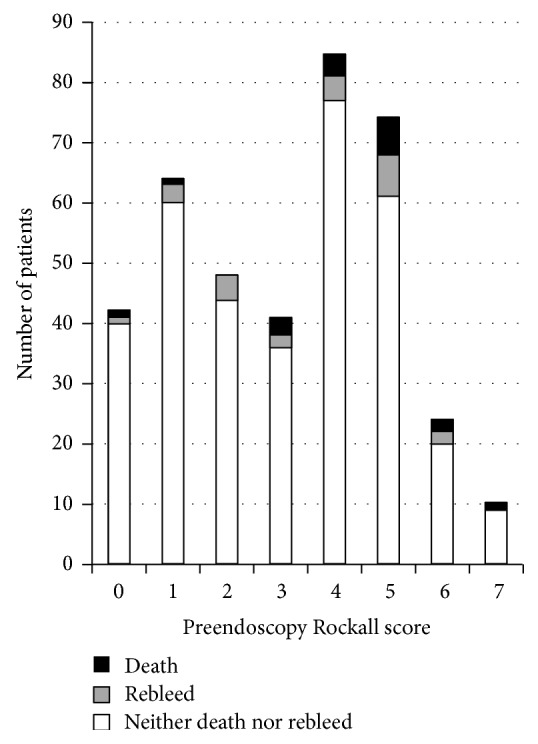
Preendoscopy Rockall score (PERS) for 388 consecutive patients admitted with upper gastrointestinal haemorrhage over 5 years (2006–2011). The overall 30-day mortality was 4.6% with a 14-day rebleed rate of 5.9%.

A PERS of 0 was seen in 42 cases (10.8%). Of these, 15 required an intervention (10 a blood transfusion, 4 endoscopic therapy, and one both). One died within 30 days and 1 experienced a rebleed within 14 days. The frequency of PERS and need for intervention is shown in Figure 2 and the secondary outcomes according to PERS are in Figure 3.

Sensitivity, specificity, and positive and negative predictive values for a PERS of zero are shown in Table 3. To enable a more detailed comparison, equivalent details are given for GBS scores of 0, ≤1, ≤2, and ≤2 with age modification given in Table 4. Compared to males, females were significantly more likely to require intervention (OR = 1.73, CI: 1.12–2.68: *p* = 0.01).

The PERS was not significantly related to either the need for any intervention (*p* = 0.13) or the risk of rebleeding (*p* = 0.35). However, it was significantly (*p* < 0.01) related to 30-day mortality with an odds ratio of 1.46 (CI: 1.11–1.92) for each Rockall point increase. Also, NSAID/aspirin use was significantly associated with a decreased 30-day mortality rate (OR 0.22, CI: 0.08–0.60: *p* < 0.005).

## 4. Discussion

We identified 424 patients over an almost 5-year period admitted with an upper GI haemorrhage. This equates to 47.9/100,000 pa, which is within the expected range (20–60/100,000) for such admissions; that is, although this study was retrospective it is likely that we have identified the majority of patients presenting with UGIH.

PERS is significantly correlated with 30-day mortality, but not with risk of intervention or rebleeding. Even when a cut-off of 0 was used it had lower sensitivity (91.7%) in identifying low risk patients not requiring endoscopic intervention compared to GBS [17]. These results are consistent with most previous studies [10, 12, 22] where GBS outperforms PERS with respect to both sensitivity and NPV. Using a GBS < 1 without age modification we have reported sensitivity and NPV of 100% and 49%. The Rockall score was originally designed to stratify patients according to mortality risk but was not designed and should not be used on the basis of these results to stratify patients into low risk category for outpatient management.

Our study population had a smaller proportion of cases stratified as low risk despite using a higher GBS to define low risk than many studies. Previous European studies have found the proportion of low risk patients to be 4.9–34.2% [6, 12, 13, 15, 23] and studies from Asia to be 7.9–13% [10, 11]. Despite this, only 46.6% of our study participants required any intervention, which is not abnormally high, with significant heterogeneity of intervention rates noted internationally [6, 10–13, 23].

Using GBS, the low prevalence of low risk patients may partly result from over three-quarters of cases in our study presenting with melaena, for which one point is assigned in the GBS. Using PERS, age over 60 years is allocated 1 and over 80, 2 points. As the median age was 73 years, many patients have had scores greater than zero based on age alone.

Another possible explanation is that low risk patients are not being managed according to hospital protocol. Admission is advised for all patients with UGIH regardless of severity. If these patients are being referred to outpatient services at the discretion of primary care practitioners or being discharged from the emergency department without admission then this would reduce the numbers of low risk patients being admitted and the proportion of high risk cases would be overrepresented. This is a limitation of our study as it is retrospective and cannot capture such patients. It is more likely that low rather than high risk patients are not admitted.

Other studies have not quoted the numbers of patients in their area being assessed by primary care physicians as low risk and referred as outpatients. We do know that 193 patients during the 5 years of the study period here had a gastroscopy as an outpatient for UGIH suggesting that triage by primary care and referral as an outpatient of perceived low risk cases do contribute to the low percentage of low risk patients in our cohort.

The basic demographics of our study population are similar to most other studies, with a higher incidence in males (55% male) despite an older median age of 74.3 years [17]. Ethnicity data was similar to that found in the most recent national census in 2006 [24] although Maori were underrepresented making up 2% of our study population compared to 7.2% in the census.

The rate of rebleeding was 6.0% in our patient group which is relatively low (6–14.6% found previously) [6, 10, 25]. This could potentially be a reflection on variations in disease prevalence, for example, a lower incidence of variceal haemorrhage.

37.8% of patients received a transfusion with 81.2% of patients who required intervention having a blood transfusion. The average proportion of patients transfused varies significantly from 26 to 75.3% [5, 10–12, 26], though rates in New Zealand have previously been reported at a similar level of 46.6% [27].

The mortality rate at 4.6% was lower than expected [5, 6, 27]. This may be because we excluded inpatient UGIH, a group with higher mortality, for example, 26% versus 7% for outpatient UGIH in the NHS National Comparative Audit of Blood Transfusion in 2007 [6]. Also there were fewer patients with variceal bleeding in our cohort, a condition associated with higher mortality.

Those admitted while taking NSAIDs/aspirin had a greatly reduced 30-day mortality (OR = 0.23, *p* < 0.005). Although we might expect NSAID usage to be the cause of UGIH in some cases, there are several possible explanations for reduced 30-day mortality. The most likely explanation is that as aspirin made up the majority of patients taking NSAIDs (75.2%), this risk reduction may represent protection against other causes of mortality such as myocardial infarction and stroke. Mortality following UGIH is more commonly related to comorbidity than the bleeding itself.

The commonest endoscopic therapy employed was injection of adrenaline followed by injection of ethanolamine (Table 2) to manage ulcers with stigmata of recent haemorrhage. It has been shown to be safe and effective therapy to reduce risk of rebleeding. A recent Cochrane meta-analysis has shown that adrenaline followed by either injection of a second agent or thermal or mechanical modality is more effective than adrenaline injection alone but has not shown any superiority of thermal or mechanical device versus use of a second injectable agent [28].

The incidence of variceal haemorrhage is lower than many previously reported studies. The alcohol intake in New Zealand is traditionally lower than that seen in the United States and much of Europe, although rates of alcohol related illness are higher in Maori. We have a low Maori and Pacific Island population in lower South Island and hence the incidence of alcoholic cirrhosis is particularly low. We speculate also that obesity is relatively uncommon reducing the incidence of nonalcoholic fatty liver disease. The OECD statistics for death from cirrhosis and chronic liver disease in NZ showed 115 deaths in 2010 for a population near 4 million; that is, it is uncommon [29].

In summary, we have shown that preendoscopy Rockall score cannot be used to identify safely those suitable for outpatient endoscopy despite identifying more than twice the number of low risk patients as GBS presenting with upper gastrointestinal bleeding to a New Zealand teaching hospital. There are fewer patients identified as low risk than seen in European and North American studies and this may be due to a relatively large number of patients with UGIH being referred as outpatients by primary care and the emergency department, presumably having been informally risk-stratified. PERS can be used to identify patients at higher risk of dying within 30 days of admission but whether this can be used to intensify their care and improve outcomes remains to be proven.

## Figures and Tables

**Table 1:** 

Clinical feature		
Presenting symptom	Melaena	299 (77.1%)
	Fresh haematemesis	67 (17.3%)
	Coffee ground haematemesis	96 (24.7%)
	Unspecified haematemesis	14 (3.6%)

Syncope		46 (11.9%)

Symptom duration	<24 hours	205 (52.8%)
	1–7 days	120 (30.9%)
	>7 days	63 (16.2%)

Admission medication	PPI	175 (45.1%)
	NSAID (including aspirin)	286 (73.7%)
	Anticoagulant	55 (14.1%) (50 warfarin)

**Table 2:** Outcomes and interventions.

		Number of cases (%)
Total		388 (100)

Outcomes	Intervention required	181 (46.6%)
	Death within 30 days	18 (4.6%)
	Rebleeding within 14 days	23 (5.9%)

Interventions	Blood transfusion	147 (37.9%)
	Endoscopic intervention	75 (19.3%)
	Surgery	8 (2.1%)

Endoscopic intervention by type	Injection (adrenaline followed by ethanolamine)	55 (14.2%)
	Endoclip	16 (4.1%)
	Banding	5 (1.3%)
	Argon plasma coagulation	3 (0.8%)
	Injection (unspecified)	1 (0.3%)

Data are from 388 patients admitted with upper gastrointestinal haemorrhage over 5 years (2006–2011).

**Table 3:** Sensitivity and specificity of a preendoscopy Rockall score of zero.

	Requirement for intervention (95% CI)	Rebleeding within 14 days (95% CI)	Death within 30 days (95% CI)
Sensitivity	91.7% (86.7–95.3)	95.7% (78.1–99.9)	94.4% (72.7–99.9)
Specificity	13.0% (8.8–18.4)	11.2% (8.2–14.9)	11.1% (8.1–14.7)
Positive predictive value	48.0% (42.6–53.4)	6.4% (4.0–9.5)	4.9% (2.9–7.8)
Negative predictive value	64.3% (48.0–78.4)	97.6% (87.4–99.9)	97.6% (87.4–99.9)

Data are from 388 patients admitted with upper gastrointestinal haemorrhage over 5 years (2006–2011). Interventions include blood transfusion and endoscopic or surgical intervention.

**Table 4:** Sensitivity and specificity of various Glasgow Blatchford scores for primary and secondary outcomes.

		Requirement for intervention	Rebleeding within 14 days	Death within 30 days
		(95% CI)	(95% CI)	(95% CI)
	All ages			
GBS = 0	Sensitivity	100% (97.0–100)	100% (78.9–100)	100% (74.0–100)
	Specificity	2.4% (0.8–5.5)	1.4% (0.5–3.2)	1.4% (0.4–3.1)
	Positive predictive value	47.3% (42.2–52.4)	6.1% (3.8–8.9)	4.7% (2.8–7.3)
	Negative predictive value	100% (35.9–100)	100% (35.9–100)	100% (35.9–100)

	All ages			
GBS ≤ 1	Sensitivity	100% (97.0–100)	100% (78.9–100)	100.0% (74.0–100)
	Specificity	6.8% (3.8–11.1)	3.8% (2.1–6.4)	3.8% (2.1–6.3)
	Positive predictive value	48.4% (43.2–53.6)	6.2% (3.9–9.1)	4.8% (2.9–7.5)
	Negative predictive value	100% (68.1–100)	100% (68.1–100)	100.0% (68.1–100)

	All ages			
GBS ≤ 2	Sensitivity	97.8% (94.4–99.4)	100% (78.9–100)	94.4% (72.7–99.9)
	Specificity	11.1% (7.2–16.2)	7.4% (4.9–10.6)	7.0% (4.6–10.1)
	Positive predictive value	49.0% (43.8–54.3)	6.4% (4.1–9.4)	4.7% (2.8–7.4)
	Negative predictive value	85.2% (66.3–95.8)	100% (81.7–100)	96.3% (81.0–99.9)

	<70 years old			
GBS ≤ 2	Sensitivity	98.9% (96.1–99.9)	100.0% (78.9–100)	94.4% (72.7–99.9)
	Specificity	9.7% (6.0–14.5)	6.0% (3.8–9.0)	5.7% (3.5–8.6)
	Positive predictive value	48.9% (43.7–54.1)	6.3% (4.0–9.3)	4.6% (2.7–7.3)
	Negative predictive value	90.9% (70.8–98.9)	100.0% (78.1–100)	95.5% (77.2–99.9)

Data are from 388 patients admitted with upper gastrointestinal haemorrhage over 5 years (2006–2011). Glasgow Blatchford score (GBS) was determined retrospectively. Specificity and sensitivity for various scores, previously shown to delineate low likelihood of requiring intervention, were defined. Primary outcomes are endoscopic or surgical therapy or blood transfusion and secondary outcomes are rebleed within 14 days or death within 30 days.
